# Urinary tract infection caused by a small colony variant form of capnophilic *Escherichia coli* leading to misidentification and non-reactions in antimicrobial susceptibility tests

**DOI:** 10.1186/s13756-018-0438-6

**Published:** 2018-11-20

**Authors:** Yu Jin Park, Nguyen Le Phuong, Naina Adren Pinto, Mi Jeong Kwon, Roshan D’Souza, Jung-Hyun Byun, Heungsup Sung, Dongeun Yong

**Affiliations:** 1 Department of Laboratory Medicine and Research Institute of Bacterial Resistance, Severance Hospital, Yonsei University College of Medicine, 50-1 Yonsei-ro, Seodaemun-gu, Seoul, 03722 Republic of Korea; 2 J.Craig Venter Institute (JCVI), 9605 Medical Center Dr #150, Rockville, MD 20850 USA; 3 Department of Laboratory Medicine, Asan Medical Center, University of Ulsan College of Medicine, 88 Olympic-ro 43-gil, Songpa-gu, Seoul 05505 Republic of Korea

**Keywords:** Small colony variant, Capnophilic, *Escherichia coli*, Misidentification

## Abstract

**Background:**

Small colony and capnophilic variant cases have been separately reported, but there has been no reports of their simultaneous presence in one isolate. We report a case of *Escherichia coli* with coexpressed small colony and capnophilic phenotypes causing misidentification in automated biochemical kits and non-reactions in antimicrobial susceptibility test cards.

**Case presentation:**

An 86-year-old woman developed urinary tract infection from a strain of *Escherichia coli* with SCV and capnophilic phenotypes in co-existence. This strain did not grow without the presence of CO_2_, and therefore proper identification from automated system was not possible. 16 s rRNA sequencing and matrix-assisted laser desorption/ionization time-of-flight mass spectrometry was able to identify the bacteria.

**Conclusion:**

As these strains do not grow on culture parameters defined by CLSI or on automated systems, proper identification using alternative methods are necessary.

## Background

Small colony variants (SCV) can be defined as a naturally occurring sub-population of bacteria characterized by their reduced colony size and distinct biochemical properties [1]. Capnophilic *E. coli*, which thrive in the presence of high concentrations of carbon dioxide, have rarely been reported [2, 3]. SCV and capnophilic variant cases have never been reported in co-existence. Herein, we report the first case of *E. coli* with coexpressed SCV and capnophilic phenotypes isolated from a urinary tract infection.

## Case report

An 86-year-old woman visited our hospital with foamy urine and foul odor. Urinalysis showed many WBCs (163.7 WBCs/μL) and bacteria (11,343.7 bacteria/uL), and positivity for nitrite. Gram-negative coccobacilli were revealed upon microscopic examination. The sample was cultured on sheep blood agar plate (BAP) and MacConkey agar plates at 35 °C in a 5% CO_2_ atmosphere for 24 h. After one day of incubation, > 100,000 CFU/ml of pinpoint Gram-negative colonies grew on the BAP with 10,000 CFU/ml of Gram-positive cocci. After isolation of pinpoint colonies and another 24-h incubation, the pinpoint Gram-negative colonies were irregularly divided into large colonies and pinpoint SCV colonies on BAP (Table 1).

**Table 1:**
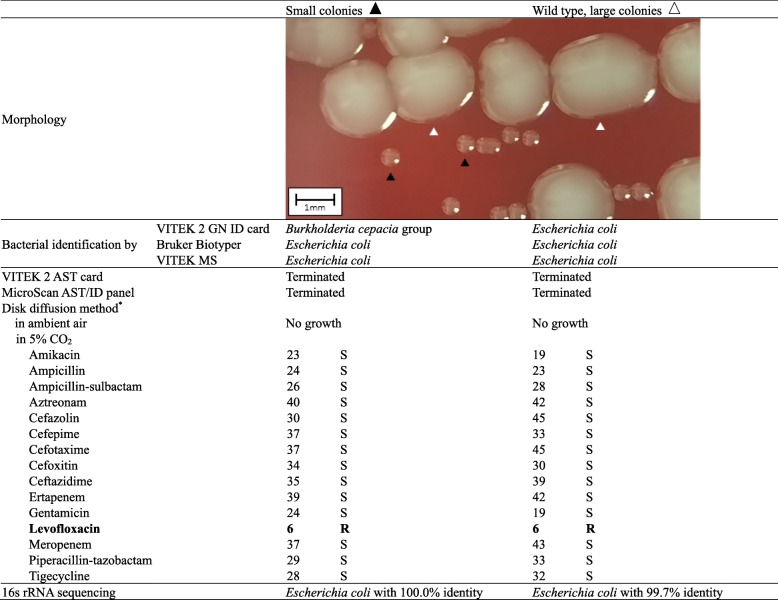
Bacterial identification and antimicrobial susceptibility testing results

*Disk diffusion method results are given as measured zone diameters [8] and interpretive category. *S* susceptible, *R* resistant

While the VITEK 2 system (bioMerieux, Durham, USA) identified the pinpoint colony as *Burkholderia cepacia* group, the Bruker Biotyper (Bruker Daltonics, Leipzig, Germany) and VITEK MS (bioMerieux, Marcy-l’Étoile, France) matrix-assisted laser desorption/ionization time-of-flight mass spectrometry (MALDI-TOF MS) systems identified both colonies as *E. coli.* The 16 s rRNA sequencing concluded both isolates were *E. coli*. As automated systems in an ambient air were unable to grow capnophilic SCVs, antimicrobial susceptibility testing profile was determined through disk diffusion method [4]. With the exception of levofloxacin resistance, bacteria was susceptible to all other antimicrobials. From these findings, we concluded that this isolate was CO_2_-dependent and had the ability to revert to its natural large form in the presence of CO_2_.

Whole genome sequencing analysis by the MiSeq® system (Illumina, San Diego, USA) was performed to inspect assumed genes that contained previously-reported causative mutations for the *E. coli* SCV phenotype (*hemB*, *menC*, and *lipA* gene) [1, 5], but no genetic mutational variations were observed between the two strains. The *yadF* gene was not present in either strain, which is consistent with previous reports about capnophilic *E. coli* strains [6].

## Discussion

The first *E. coli* SCV was reported in 1931, but there have been only few reports from clinical specimens [7–9]. Interestingly, this SCV strain was also capnophilic. The bacterial growth for reported capnophilic *E. coli* strains formed either large colonies in the presence of CO_2_ or no colonies in the absence of CO_2_ [2, 3]. To the best of our knowledge, this is the first report of *E. coli* with coexpressed SCV and capnophilic phenotype. Fortunately, this strain was susceptible to all other antimicrobials with the exception of levofloxacin, and therefore did not cause any severe outcome clinically. However, if this strain was to acquire drug resistance in the future, it is diagnostically crucial not to misidentify or neglect such strain for proper therapeutic purposes.

Additional criteria including CO_2_ conditions are needed because CLSI guidelines defining incubation conditions for *Enterobacteriaceae* involve 35 °C ambient air [4], which are unsuitable for growing capnophilic SCVs. We advise that all urine cultures should be incubated in an environment containing 5% CO_2_ to avoid overlooking of such strains. Proper identification using alternative methods such as MALDI-TOF MS systems are necessary for these capnophilic strains.

## Abbreviations

BAP: Blood agar plate
MALDI-TOF MS: Matrix-assisted laser desorption/ionization time-of-flight mass spectrometry
SCV: Small colony variants
